# Using ToxCast™ Data to Reconstruct Dynamic Cell State Trajectories and Estimate Toxicological Points of Departure

**DOI:** 10.1289/ehp.1409029

**Published:** 2015-10-16

**Authors:** Imran Shah, R. Woodrow Setzer, John Jack, Keith A. Houck, Richard S. Judson, Thomas B. Knudsen, Jie Liu, Matthew T. Martin, David M. Reif, Ann M. Richard, Russell S. Thomas, Kevin M. Crofton, David J. Dix, Robert J. Kavlock

**Affiliations:** 1 National Center for Computational Toxicology, Office of Research and Development, U.S. Environmental Protection Agency, Research Triangle Park, North Carolina, USA; 2 Department of Statistics, North Carolina State University, Raleigh, North Carolina, USA; 3 Oak Ridge Institute for Science Education (ORISE), U.S. Department of Energy, Oak Ridge, Tennessee, USA; 4 Department of Biological Sciences, North Carolina State University, Raleigh, North Carolina, USA

## Abstract

**Background:**

High-content imaging (HCI) allows simultaneous measurement of multiple cellular phenotypic changes and is an important tool for evaluating the biological activity of chemicals.

**Objectives:**

Our goal was to analyze dynamic cellular changes using HCI to identify the “tipping point” at which the cells did not show recovery towards a normal phenotypic state.

**Methods:**

HCI was used to evaluate the effects of 967 chemicals (in concentrations ranging from 0.4 to 200 μM) on HepG2 cells over a 72-hr exposure period. The HCI end points included p53, c-Jun, histone H2A.x, α-tubulin, histone H3, alpha tubulin, mitochondrial membrane potential, mitochondrial mass, cell cycle arrest, nuclear size, and cell number. A computational model was developed to interpret HCI responses as cell-state trajectories.

**Results:**

Analysis of cell-state trajectories showed that 336 chemicals produced tipping points and that HepG2 cells were resilient to the effects of 334 chemicals up to the highest concentration (200 μM) and duration (72 hr) tested. Tipping points were identified as concentration-dependent transitions in system recovery, and the corresponding critical concentrations were generally between 5 and 15 times (25th and 75th percentiles, respectively) lower than the concentration that produced any significant effect on HepG2 cells. The remaining 297 chemicals require more data before they can be placed in either of these categories.

**Conclusions:**

These findings show the utility of HCI data for reconstructing cell state trajectories and provide insight into the adaptation and resilience of in vitro cellular systems based on tipping points. Cellular tipping points could be used to define a point of departure for risk-based prioritization of environmental chemicals.

**Citation:**

Shah I, Setzer RW, Jack J, Houck KA, Judson RS, Knudsen TB, Liu J, Martin MT, Reif DM, Richard AM, Thomas RS, Crofton KM, Dix DJ, Kavlock RJ. 2016. Using ToxCast™ data to reconstruct dynamic cell state trajectories and estimate toxicological points of departure. Environ Health Perspect 124:910–919; http://dx.doi.org/10.1289/ehp.1409029

## Introduction

A major focus in public health has been to understand and limit potential adverse health effects of chemicals. However, despite an expectation of safety by the general public, there are tens of thousands of chemicals in commerce that have been evaluated on the basis of closely related analogs but that lack chemical-specific toxicity information (Judson et al. 2009). This lack of toxicity information has led to national and international efforts to use *in vitro* high-throughput screening (HTS) methods to collect data on biochemical and cellular responses following chemical treatment *in vitro* (Kavlock et al. 2009; Attene-Ramos et al. 2013). A key element of toxicity testing in the 21st century [National Research Council Committee on Toxicity Testing and Assessment of Environmental Agents (NRC) 2007; Boekelheide and Andersen 2010] is conceptually organizing HTS data into pathways that, when sufficiently perturbed, lead to adverse outcomes. One challenge associated with this new vision has been the assessment of “tipping points” beyond which pathway perturbations invoke a lasting change that could ultimately lead to an adverse effect.

The present study is part of the U.S. Environmental Protection Agency’s (EPA’s) ToxCast™ project, which aims to develop *in vitro* screens to identify potentially hazardous substances for further targeted testing (Kavlock et al. 2012). We used high-content imaging (HCI) (Giuliano et al. 2006), which applies automated image analysis techniques to capture multiple cytological features using fluorescent labels, to measure the concentration-dependent dynamic changes in the state of HepG2 cells. Although they are not fully metabolically capable, HepG2 cells can undergo continuous proliferation in culture and have a demonstrated capacity to predict hepatotoxicity of pharmaceutical compounds with good sensitivity and specificity (O’Brien et al. 2006; Abraham et al. 2008). We used computational tools to deconvolute HCI responses into cell-state trajectories and to analyze them for their propensity to recover to normal (basal) conditions over the test period. The critical concentrations associated with nonrecoverable cellular trajectories were determined, where possible, and compiled into a novel chemical classification scheme. We discuss how these “tipping points” in the function of cellular systems might be used to define a point of departure for risk-based prioritization of environmental chemicals.

## Methods

### Cell Culture

HepG2 cells were obtained from American Type Culture Collection (ATCC) and used before passage 20. Cells were maintained and expanded in complete media [10% fetal bovine serum (FBS) in Minimum Essential Medium with Earle’s Balanced Salt Solution (MEM/EBSS) supplemented with penicillin/streptomycin, L-glutamine, and non-essential amino acids]. Cell culture reagents were obtained from VWR International. HepG2 cells were harvested by trypsinization and plated at different densities in 25 μL of culture medium, depending on incubation time, in clear-bottom, 384-well microplates (Falcon #3962) that were coated with rat tail collagen I. The cells were incubated overnight to allow attachment and spreading.

### Chemical Treatments

HepG2 cells were treated with 967 chemicals from ToxCast™ Phase I and Phase II libraries (U.S. EPA 2014). Cells were treated with dimethyl sulfoxide (DMSO) as a solvent control at a final concentration of 0.5% v/v or with compounds in DMSO with a resulting final DMSO concentration of 0.5% v/v. Compound treatment was done at concentrations of 0.39, 0.78, 1.56, 3.12, 6.24, 12.5, 25, 50, 100, and 200 μM in duplicate on each plate. Cells were treated with ToxCast™ Phase I compounds for 1, 24, and 72 hr and ToxCast™ Phase II compounds for 24 and 72 hr only. Carbonyl cyanide *m*-chlorophenylhydrazone (CCCP) and taxol were used as positive controls for mitochondrial function and cytoskeletal stability, respectively; DMSO served as the negative control for this experiment.

### Cell Staining and Fluorochroming

Cells were fixed by the direct addition of 50 μL formaldehyde in Hank’s Balanced Salt Solution (HBSS) to a final concentration of 3.7%. After incubation in the fixation medium for 30 min at room temperature (293–298 K), cells were rinsed twice with HBSS and treated with cell permeabilization buffer (16 μL of 0.5% Triton X-100) for 10 min at room temperature (293–298 K) before labeling. For mitochondrial membrane potential and mitochondrial measurements, pre-fixed cells were incubated with 50 μL of MitoTracker® Red CMXRos (Invitrogen) at a concentration of 250 nM for 30 min before fixation. In the remaining cases, post-fixed cells were labeled by incubation with a multiplexed mixture of primary antibodies in HBSS for 60 min at room temperature (293–298 K) to detect immunoreactivity of c-Jun (1:500), phospho-histone H3 (1:100), phospho-histone H2A.x (1:200), p53 (1:400), α-tubulin (1:200) and Hoechst 33342 (2 μg/mL). Cells were labeled for multiplexed imaging on two separate plates: *a*) Hoechst 33342, MitoTracker® Red, phospho-histone H3, and α-tubulin, and *b*) Hoechst 33342, phospho-histone H2A.x, and c-Jun. A final rinse with HBSS (50 μL) was performed before analysis. The primary and secondary antibodies for the proteins were phospho-histone H3 (rabbit anti-phospho-histone H3 and FITC-donkey anti-rabbit IgG), phospho-histone H2A.x (mouse anti-phospho-histone H2A.x and FITC-donkey anti-mouse IgG), c-Jun (rabbit anti-phospho-c-Jun and Cy3-donkey anti-rabbit IgG), p53 (sheep anti-p53 and Cy5-donkey anti-sheep IgG), α-tubulin (mouse anti-α-tubulin and Cy5-donkey anti-mouse IgG). These antibodies are available as the CellCiphr HepG2 assay kit (Millipore).

### Image Acquisition, Analysis and Feature Extraction

Digital images of each well were captured using a Cellomics ArrayScan VTI (Thermo Scientific Cellomics™) (0.8 NA objective, 0.63× optical coupler, and XF-93 filter set) at 20× magnification. The images were acquired using the autofocus feature of the ArrayScan instrument, which entails the following steps. First, the camera focuses on channel 1 (Hoechst 33342), where nuclei are identified. Second, a *Z* offset of 1 μm is used for capturing mitochondria (MitoTracker® Red). Third, a *Z* offset of –2 μm is used for capturing the cytoskeleton (tubulin). Six digital images were captured in each well and analyzed using BioApplication software, which was provided with the instrument. All images were analyzed using the Compartmental Analysis and Cell Cycle Analysis BioApplication software from Cellomics™. The Cell Cycle BioApplication software (Cellomics™ 2007a) used the nuclear stain to identify valid cells, to measure nuclear diameter, and to quantify DNA content. These features were used to calculate the average nuclear size, cell cycle arrest (ratio of 2N/4N), and cell number. The Compartmental Analysis BioApplication software module (Cellomics™ 2007b) was used to measure the average cell intensities for c-Jun phosphorylation, p53 protein activation, phospho-histone H2A.x activation, mitochondria, and α-tubulin. The average intensity of mitochondria was used to define mitochondrial membrane potential, and the total intensity was used to define mitochondrial mass. Data from cellular features measured in the nucleus were excluded for wells where there was a significant decrease in nuclear size and brightness. Detailed documentation about the algorithms and parameters used by the BioApplication software for this analysis are available upon request. Cellular features were aggregated at the well level to quantify the following end points: p53 activation, c-Jun activation (stress kinase), phospho-histone H2A.x (DNA damage produced by oxidative stress), phospho-histone H3 (mitotic arrest), α-tubulin (microtubules), mitochondrial membrane potential, mitochondrial mass, cell cycle arrest, nuclear size, and cell number. Table S1 summarizes the relationships between cellular end points, stains/fluorochromes, the BioApplication software, and the specific algorithms used for extracting cell-level features. The raw image data (captured by the ArrayScan VTI) and well-level data for all chemical treatment concentrations, time points, and stains/fluorochromes were stored in a freely available custom database (https://www.epa.gov/chemical-research/downloadable-computational-toxicology-data). Representative HCI images captured 1, 24, and 72 hr after treatment with CCCP, taxol, butachlor, fludioxonil, and fluazinam are shown in Figures S1(a), S1(b), S1(c), S1(d), and S1(e), respectively.

### Data Processing and Normalization

Concentration response data from the HCI experiment were smoothed and normalized for every chemical, end point, and time. The raw concentration responses were smoothed using a Hamming window (Blackman and Tukey 1958) of length 7. The raw concentration-dependent responses for the reference chemicals, CCCP and taxol, are shown in Figures S2(a) and S2(b), respectively. The raw time-dependent responses for CCCP and taxol are shown in Figures S2(c) and S2(d), respectively. Additional examples of raw smoothed concentration- and time-dependent responses for fludioxonil, fluazinam, and butachlor are shown in Figure S3. Next, the smoothed data (*r*) for end points measured on each plate were normalized to the median response (r*) to calculate perturbations as the logarithm (base 2) of fold change values. The normalized changes (x = log_2_ r/r*) were also standardized (z = (x – x*)/σ*_x_*) to evaluate the importance of perturbations (where σ*_x_* is the standard deviation of *x*). The lowest effect concentration (LEC) for each chemical and end point was calculated as the concentration that produced a fold change perturbation at least one standard deviation (i.e., σ*_x_* = 1) above or below the median value. An absolute perturbation > one standard deviation was called a “hit” (i.e., |σ*_x_*| > 1). The LEC was estimated by numerically solving for: |*z*| = 1 (the minimum value was selected if there were multiple solutions). The efficacy was measured as maximum positive or negative value of *x*.

### System Trajectory and Dynamics

**Figure 1:**
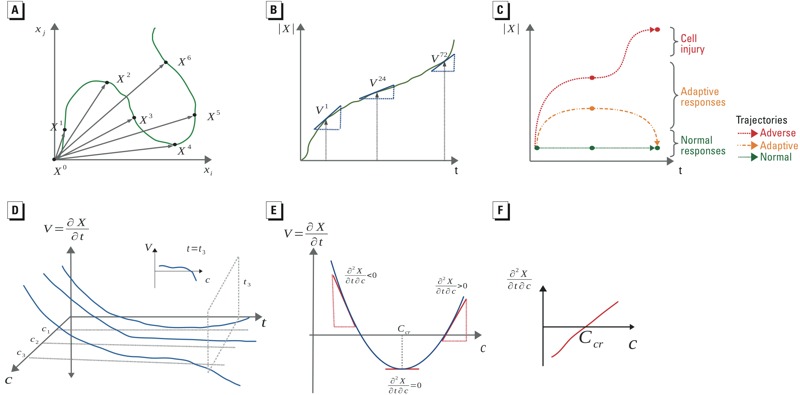
Hypothetical dynamic system perturbations as trajectories and calculation of tipping points. (*A*) The green curve depicts a hypothetical trajectory across observations at time t (*X^t^*) shown on the basis of two endpoints (*x_i_* and *x_j_*). (*B*) The perturbation velocity (*V*) is calculated as the derivative of the scalar perturbation (|**X** *|*) with respect to time (shown in green). (*C*) Three different types of trajectories are shown using |**X** *|*: trajectories that describe the normal behavior of the system (shown in green); adaptive trajectories, which include some perturbation of the system state followed by recovery (shown in orange); and adverse trajectories that show initial adaptive responses followed by lack of recovery at later times (shown in red). (*D*) The relationship between the velocity, concentration, and time is given by a continuous surface, *V = f*(*c,t*). (*E*) The rate of change of velocity with respect to concentration is given by ∂*_c_V* = ∂*V*/∂*c* = ∂^2^ *X*/∂t∂*c*. (*F*) Solving ∂*_c_V = 0* gives the critical concentration, *C_cr_*.

Each concentration and duration of chemical treatment produced a system perturbation (***X***), which was represented by the vector: ***X*** = (*x_sk_, x_os_, x_p53_, x_mt_, x_mm_, x_mmp_, x_ma_, x_cca_, x_ns_, x_cn_*) (where the subscripts *sk*, *os*, *p53*, *mt*, *mm*, *mmp*, *ma*, *cca*, *ns*, and *cn* denote stress kinase, oxidative stress, p53, microtubules, mitochondrial mass, mitochondrial membrane potential, mitotic arrest, cell cycle arrest, nuclear size, and cell number, respectively). The vector perturbation was also summarized by a scalar magnitude (*X*), which was calculated as the Euclidean norm [*X* = *|* ***X***| = (Σ*x_i_^2^*)^1/2^]. We defined a trajectory (*T*) as the dynamic response of the system to a chemical concentration as a temporal sequence of scalar perturbations, *T* = (*X^0^, X^1^, X^2^, .., X^t^,.., X^n^*). The scalar system perturbation was assumed to be continuous across concentration and time [*X = f*(*c*,*t*)] and was estimated from experimental data [we assumed that the system was unperturbed at *t* = 0, i.e., *f*(*c*,*0*) *= 0*]. The velocity of the system (*V*) was defined as the rate of change of the scalar system perturbation (*V = ∂X/∂t*) and was calculated as the slope of *X* with respect to time *t*. At a given time point, normal, recovering, and nonrecovering trajectories are defined by: *V = 0*, *V < 0* and *V > 0*, respectively. The concepts of system trajectory, velocity, and recovery are illustrated in Figures 1A, B, and C, respectively.

### Quantifying System Recovery Across Concentrations

We assumed that *V* formed a two-dimensional surface from which the recovery of the system could be analyzed at any time (*t*) across concentrations (*c*) (Figure 1D). Consider a hypothetical parabolic relationship between *V* and *c* at a fixed time (shown in Figure 1E). At low concentrations, *V* is positive, which suggests that the system perturbation is increasing. As the concentration increases, *V* decreases until it reaches a minimum and then begins to increase. These trends can be summarized by the rate of change of *V* with respect to concentration (∂_c_ *V* = ∂*V*/∂*c* = ∂^2^ *X*/∂t∂*c*), which can have three possible values: *a*) ∂_c_ *V < 0* for concentrations that produce recovery, *b*) ∂_c_ *V > 0* for concentrations that do not produce recovery, and *c*) ∂_c_ *V = 0* for the concentration corresponding to the critical point for system recovery. For each chemical, the empirical relationship between ∂_c_ *V* and different treatment concentrations at 72 hr was estimated by B-spline interpolation and numerically solved for ∂_c_ *V = 0* to calculate the critical concentration (denoted as *C*_cr_). After resampling 50 subsets of the concentration–velocity pairs for each chemical, ∂_c_ *V* was fitted and solved for ∂_c_ *V = 0* to construct a distribution, which was used to estimate the 95% confidence interval for *C*_cr_. We also recorded the trends in ∂_c_ *V* as a function of concentration and the frequency with which the resampled subsets produced critical points (i.e., parabolic trends in ∂_c_ *V* with maxima) or produced recovery (i.e., parabolic trends in ∂_c_ *V* with minima).

### Data Analysis Software

The data processing, storage, analysis, and visualization were performed using the freely available Python programming language (Python 2014) and associated open-source libraries. The software is freely available from the authors upon request.

## Results

### General Characteristics of Cellular Effects

The concentration–response profiles of 967 chemicals were analyzed across the 10 HCI end points and three time points to identify hits. Almost half of the chemicals (43.7% or 432/967) produced a hit for at least 1 of the 10 end points by 72 hr. Of the chemicals tested, 13.7% (132/967) changed mitochondrial membrane potential, 15.2% (147/967) altered mitochondrial mass, 22.7% (220/967) invoked oxidative stress, 9.4% (91/967) altered microtubules, 14.1% (137/967) perturbed stress kinase, 27.1% (262/967) altered p53 distribution, 17.3% (167/967) produced cell cycle arrest, 26.9% (260/967) invoked mitotic arrest, 7.7% (74/967) changed nuclear size, and 32.2% (311/967) decreased cell number. With regard to time, altered mitochondrial membrane potential (29/308) and p53 activity (14/308) were the two most frequent perturbations at 1 hr [only Phase I compounds (U.S. EPA 2014) were tested at 1 hr]; perturbations in p53 activity (168/967), mitotic arrest (157/967), and cell loss (155/967) were the most frequently observed effects at 24 hr. Finally, decrease in cell number (303/967), mitotic arrest (249/967), and p53 activity (228/967) were the most frequently observed effects at 72 hr. The LECs for all 967 chemicals across the 10 end points are provided as supplemental material in Excel Table S1.

### Cellular Perturbations

**Figure 2:**
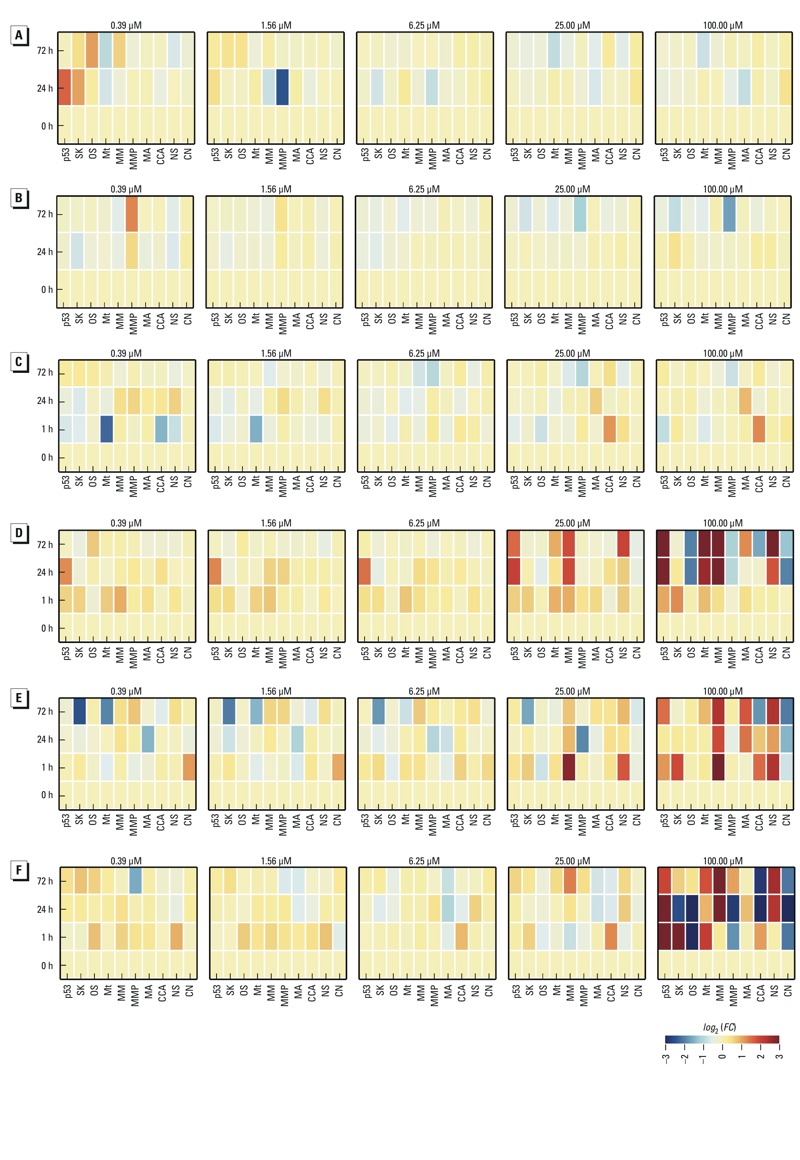
Concentration and time-dependent perturbations produced by chemicals. From top to bottom, each row of heat maps shows the perturbations produced by increasing concentrations of (*A*) octanoic acid, (*B*) dimethyl terephthalate, (*C*) chlorpyrifos-methyl, (*D*) butachlor, (*E*) dicofol, and (*F*) oxadiazon. Each heat map shows the end points (columns), time in hours (rows), and perturbations (colors) produced by each concentration (title). The end points include p53 activity, stress kinase (SK), oxidative stress (OS), microtubules (Mt), mitochondrial mass (MM), mitochondrial membrane potential (MMP), mitotic arrest (MA), cell cycle arrest (CCA), nuclear size (NS), and cell number (CN). The colors signify no effect (yellow), increase (red), and decrease (blue), and the magnitude of the changes is indicated by the color bar in the lower right corner.

Interpreting the results of the HCI experiment proved to be a complex problem because nearly half of the chemicals produced hits across multiple end points at different times. The dynamic perturbations produced by a representative subset of chemicals are shown in Figure 2 (data for all chemicals are provided as supplemental material in Excel Table S2). Each row of heat maps displays the perturbations produced by increasing concentrations (only 0.39-, 1.56-, 6.25-, 25-, and 100-μM treatments are shown) of six chemicals: Figure 2A, octanoic acid; Figure 2B, dimethyl terephthalate; Figure 2C, chlorpyrifos-methyl; Figure 2D, butachlor; Figure 2E, dicofol; and Figure 2F, oxadiazon. Each heat map shows perturbations (colors), times (rows) and end points (columns). For example, the row of heat maps in Figure 2A shows the perturbations produced by octanoic acid, which is widely used in perfumes and disinfectants. Treating HepG2 cells with 0.39 μM octanoic acid increased p53 nuclear localization (3-fold) and stress kinase activity (2-fold) at 24 hr. By 72 hr, p53 activity recovered to nearly baseline levels, but stress kinase activity remained elevated (1.4-fold). At a higher treatment concentration, 1.56 μM, octanoic acid decreased mitochondrial membrane potential (0.2-fold) at 24 hr, but mitochondrial membrane potential recovered to background levels by 72 hr. Because octanoic acid is a medium-chain fatty acid, we speculate that its effects on oxidative stress and mitochondrial function may be the result of an increase in fatty acid metabolism (Gyamfi et al. 2012).

The next row of heat maps (Figure 2B) shows the dynamic effects of dimethyl terephthalate (DMT), which is used in the production of polyesters. DMT produced a time-dependent increase (1.5-fold at 24 hr and 2.5-fold at 72 hr) in mitochondrial membrane potential and a minor decrease (0.6-fold at 24 hr and 0.7-fold at 72 hr) in nuclear size at 0.39 μM. At a treatment concentration of 100 μM, DMT caused a decrease in mitochondrial membrane potential (0.9-fold at 24 hr and 0.3-fold at 72 hr). The dual effects of DMT on MMP, increasing at 0.39 μM but decreasing at 100 μM, could be explained by transient mitochondrial hyperpolarization preceding apoptosis (Sánchez-Alcázar et al. 2000). The proportion of cells undergoing apoptosis was small, however, as there was no substantial decrease in cell number. As such, DMT exposure alters mitochondrial membrane potential with hyperpolarization at low concentrations and concentration-dependent transition to depolarization at higher concentrations. The complex mechanisms underlying such a dose-dependent transition were difficult to interpret using these HCI data alone.

The organophosphate insecticide chlorpyrifos-methyl (Figure 2C) caused microtubule disruption (0.2-fold change) at 1 hr after 0.39-μM treatments, and a decrease (0.7-fold) in cell number was observed after 72 hr for a 200-μM treatment (data not shown). Low concentrations of chlorpyrifos, which is structurally related to chlorpyrifos-methyl, in the 1-10 μM range, are known to disrupt the cytoskeleton in neurons (Flaskos et al. 2011). Chlorpyrifos is a known acetylcholinesterase inhibitor, but the relevance to cytoskeletal disruption is unclear. Unlike octanoic acid, DMT, and chlorpyrifos-methyl, butachlor (Figure 2D) produced concentration- and time-dependent perturbations across multiple end points. At a treatment concentration of 0.39 μM, butachlor increased p53 activity at 1 hr (1.5-fold) and 24 hr (2.5-fold), but p53 activity recovered to background levels by 72 hr. This temporal trend of early p53 activation followed by later recovery was observed for increasing butachlor concentrations ≤ 6.25 μM. This recovery was not evident for butachlor concentrations > 6.25 μM; hence, the p53 response was more persistent at higher concentrations. The temporal trends in mitochondrial mass tracked with p53 activity for this compound. Butachlor decreased cell number beyond 24 hr at concentrations > 100 μM. This widely used herbicide has been shown to induce DNA damage and mitochondrial dysfunction in peripheral blood mononuclear (PBMN) cells (Dwivedi et al. 2012).

Dicofol, an organochlorine pesticide, invoked concentration-dependent perturbations in mitochondrial membrane potential, p53 activity, and stress kinase at 1 and 24 hr. At 24 and 72 hr, perturbations were observed in mitochondrial mass, cell cycle arrest, nuclear size, and cell number (Figure 2E). Oxadiazon (bensulide), another organophosphate herbicide, also produced complex time- and concentration-dependent changes across all end points (Figure 2F).

### Cell-State Trajectories

We used the concept of a system trajectory to analyze the concentration- and time-dependent stress responses produced by each chemical. A trajectory describes the dynamic changes in the state of HepG2 cells in response to chemical exposure. To interpret the HCI data in terms of cell-state trajectories, we first assumed that the state of the HepG2 system could be defined by oxidative stress, stress kinase activity, mitochondrial function, cytoskeletal stability, cell cycle progression, and cell number (all of which were measured by HCI). Next, we assumed that the HCI data at each time point captured a snapshot of the state of the HepG2 system as it followed a chemical-induced trajectory. The heat maps in Figure 2A, for example, visualize trajectories for different treatment concentrations of octanoic acid. The rows in each heat map (from bottom to top) correspond to discrete snapshots of the system perturbation at successive time points (0, 1, 24, and 72 hr), and the columns in each heat map show the system state based on 10 HCI end points. We assumed that the system was initially in a “ground state” that defined the normal pattern; thus, by this definition, there were no perturbations at *t* = 0 hr.

A comparison of the trajectories produced by different chemicals (Figure 2) revealed qualitative differences across concentrations and time points. For example, the trajectories produced by 0.39 μM and 1.56 μM octanoic acid show transitory perturbations in p53 and stress kinase activities at low concentrations, but not at high concentrations (Figure 2A). In contrast, butachlor produced clearly different trajectories in temporal response profiles at concentrations ≤ 6.25 μM versus > 6.25 μM (Figure 2D). To enable quantitative analysis of trajectories in terms of both chemicals and concentrations, we developed an aggregate measure of overall system perturbation. The resulting perturbation vector (denoted as *X⃗*) describes the changes in each end point at a given time, and the scalar magnitude of *X⃗* (denoted as *X*) measures the overall perturbation of the system by combining the contributions of individual end points. When the system is in the ground state, then scalar perturbation is essentially zero (*X* = 0), but as the cellular end points change in response to chemical treatment, the scalar perturbation increases (*X* > 0).

### Trajectories and System Recovery

**Figure 3:**
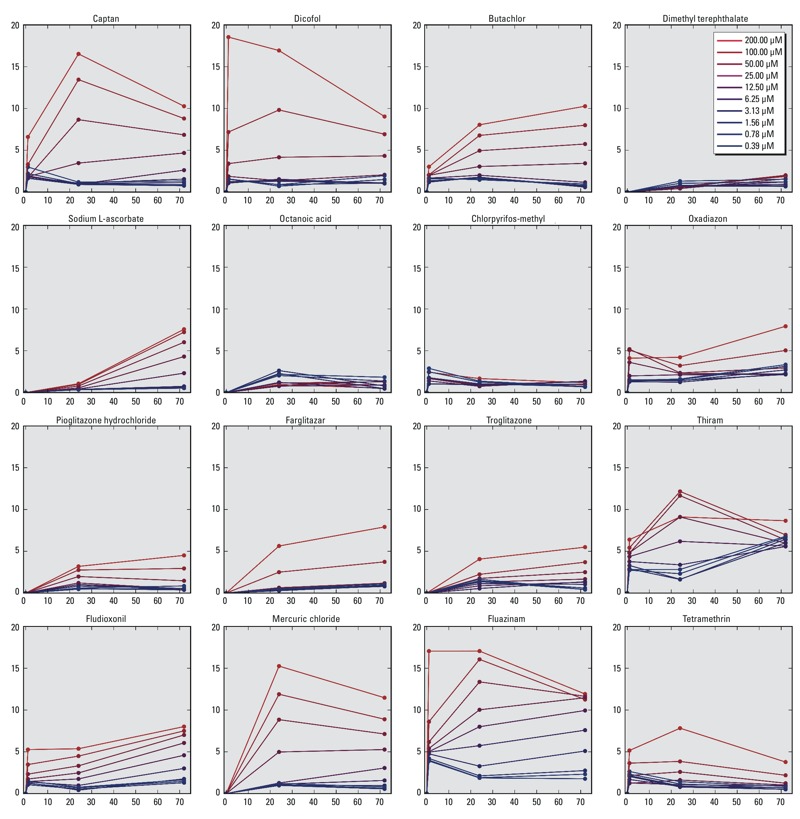
Magnitude of perturbations for trajectories produced by fixed treatment concentrations of different chemicals. Each graph shows scalar perturbations (*y*-axis) over time (*x*-axis) for multiple doses of a chemical. The colors signify treatment concentrations ranging from low (blue) to high (red).

The scalar perturbations for the trajectories were calculated for the 967 chemicals and 10 treatment concentrations to investigate concentration- and time-dependent trends. The trends for 16 representative chemicals (captan, dicofol, butachlor, dimethyl terephthalate, sodium ʟ-ascorbate, octanoic acid, chlorpyrifos-methyl, oxadiazon, pioglitazone, farglitazar, troglitazone, thiram, fludioxonil, mercuric chloride, fluazinam, and tetramethrin) are shown in Figure 3. The ordinate and abscissa of each graph in Figure 3 show the scalar perturbation (*X*) and treatment duration (hours), respectively, for each of the 16 chemicals. The treatment concentrations for each chemical are visualized as colors from low (blue) to high (red). For example, trajectories elicited by butachlor treatments showed two different temporal trends in *X*. First, butachlor treatments with concentrations < 25 μM produced an early (1 and 24 hr) increase in *X* that was followed by a later decrease (72 hr). Second, trajectories elicited by butachlor treatments ≥ 25 μM showed only an increase in *X* with time. We interpret these temporal trends as the integrated effect of chemical-induced stress, which caused *X* to deviate from the ground state, and adaptive cellular processes, which enabled the system to recover to the ground state [see Figure S1(c)]. Thus, butachlor treatments < 25 μM induced stress that dissipated with time because adaptive processes were activated in HepG2 cells that enabled system recovery. In contrast, butachlor treatments ≥ 25 μM showed a monotonic increase with time, suggesting that these higher concentrations overwhelmed the adaptive processes in HepG2 cells, and consequently, the system could not recover to its ground state.

Adaptive recovery trends were also observed for octanoic acid, captan, and dicofol (for brevity, the results for the other chemicals are not shown here, but they are available as supplemental material in Excel Table S3). The dynamic capacity of HepG2 cells to recover varied by chemical and by concentration, as illustrated by the cellular response to butachlor. Octanoic acid, however, produced smaller perturbations than butachlor, and all trajectories implied system recovery. Of the 16 representative chemicals shown in Figure 3, partial or complete recovery trajectories were evident for some compounds. The two thiazolidinediones (pioglitazone and troglitazone) also displayed similar trends, but it was difficult to compare the differences quantitatively.

### System Tipping Points

Visual inspection was useful for comparing the trends produced by different chemicals but not for quantifying concentration-dependent differences in perturbation and recovery. To further analyze the trends for each chemical, the rate of change of the scalar perturbation was calculated for the trajectories. The rate of change of the scalar perturbation across time (denoted as *V* = ∂*X*/∂t) measures the “velocity” of the system perturbation at any given point in the trajectory (described in detail in “Methods”). The velocity is negative (*V* < 0) when the system is on a trajectory that is recovering to the ground state. If the velocity is positive (*V* > 0), then the system is on a trajectory that is not recovering. The system velocity for the trajectories was thus calculated using data for *X* at 24 and 72 hr produced by all 967 chemicals and 10 treatment concentrations (results not shown). Trends in system velocity summarize the behavior of system trajectories and reveal concentration-dependent transitions that define the “tipping points” for recovery of the HepG2 cellular system. We hypothesized the broader existence of such tipping points after studying the trajectories of chemicals such as captan, dicofol, and butachlor.

To mathematically identify the tipping points of the HepG2 system using trajectories, we analyzed the relationship between perturbation velocity (*V*) and concentration (*c*). We used the rate of change of *V* with respect to concentration (denoted as, ∂_c_ *V*) to identify the concentration threshold for system recovery (see “Methods”). Like velocity, ∂_c_ *V* can have three possible values: *a*) ∂_c_ *V < 0* indicates concentrations that produce recovery, *b*) ∂_c_ *V > 0* indicates concentrations that do not produce recovery, and *c*) ∂_c_ *V = 0* signifies the critical concentration (denoted as *C*_cr_) and corresponds to the tipping point of the system. In this study, ∂_c_ *V* and *C*_cr_ were calculated for 967 chemicals using data obtained at 24 and 72 hr. We also conducted an uncertainty analysis for each chemical to evaluate confidence in trajectories and to estimate the variability in *C*_cr_ caused by experimental noise (additional details are provided in “Methods”).

**Figure 4:**
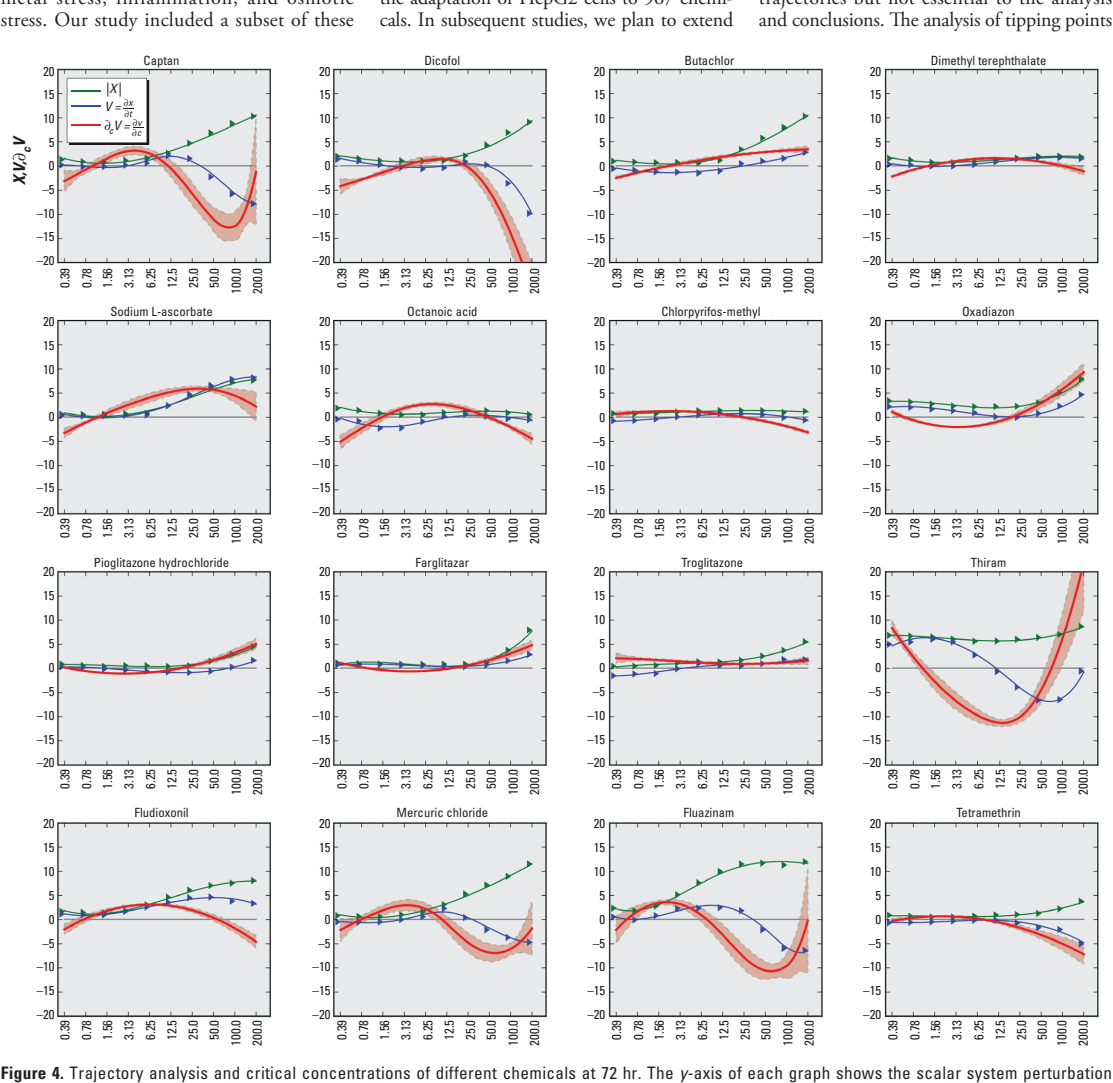
Trajectory analysis and critical concentrations of different chemicals at 72 hr. The *y*-axis of each graph shows the scalar system perturbation (*X* = green), velocity (*V* = blue) and derivative of velocity with respect to concentration (∂*_c_V* = red), and uncertainty analysis of ∂*_c_V* (light red). The *x*-axis of each graph shows the treatment concentration of the chemical (μM). Dimethyl terephthalate, sodium ʟ-ascorbate, octanoic acid, chlorpyrifos-methyl, fludioxonil, and tetramethrin produced trends in ∂*_c_V* consistent with system recovery. Butachlor, oxadiazon, pioglitazone, farglitazar, troglitazone, and thiram elicited trajectories with tipping points. Captan, mercuric chloride, and fluazinam produced complex trends in ∂*_c_V* that could be indicative of experimental noise.

The scalar perturbation (*X*), the velocity (*V*), and the derivative of velocity with respect to concentration (∂_c_ *V*) for select chemicals at 72 hr are shown in Figure 4. Two main concentration-dependent trends were used to determine the resilience of the HepG2 system to each chemical treatment. First, a subset of chemicals produced an overall decrease in ∂_c_ *V* with increasing concentrations. This trend in ∂_c_ *V* implied a recovering trajectory as invoked by, for example, DMT, sodium ʟ-ascorbate, octanoic acid, chlorpyrifos-methyl, fludioxonil, and tetramethrin. Second, a subset of chemicals elicited an overall increase in ∂_c_ *V* with increasing treatment concentrations. This increase implied a nonrecovering trajectory that contained tipping points in the cellular system identified by the condition ∂_c_ *V = 0*. Based on our analysis, butachlor, oxadiazon, pioglitazone, farglitazar, troglitazone, and thiram had critical concentrations of 2.6 ± 0.5 μM, 17.6 ± 1.2 μM, 28.4 ± 5.0 μM, 17.0 ± 2.4 μM, 4.5 ± 2.6 μM, and 69.1 ± 5.7 μM, respectively.

The resilience analysis of the HepG2 system trajectories showed that roughly one-third (334/967) of all chemicals produced recovery, another third (336/967) did not result in recovery, and the remainder (297/967) did not produce trajectories with substantial perturbations or sufficient confidence to place them in either category. Captan, mercuric chloride, and fluazinam are examples of chemicals that produced trajectories with low confidence. Visual inspection of the trajectories for these chemicals showed complex concentration-dependent trends in ∂_c_ *V* (Figure 4). Overall, 104 chemicals produced complex trends in ∂_c_ *V*, and a majority of these (71/104) produced trajectories with low confidence. Complex trends in ∂_c_ *V* could be indicative of noise and may require additional experimental data for improving confidence in the results.

**Figure 5:**
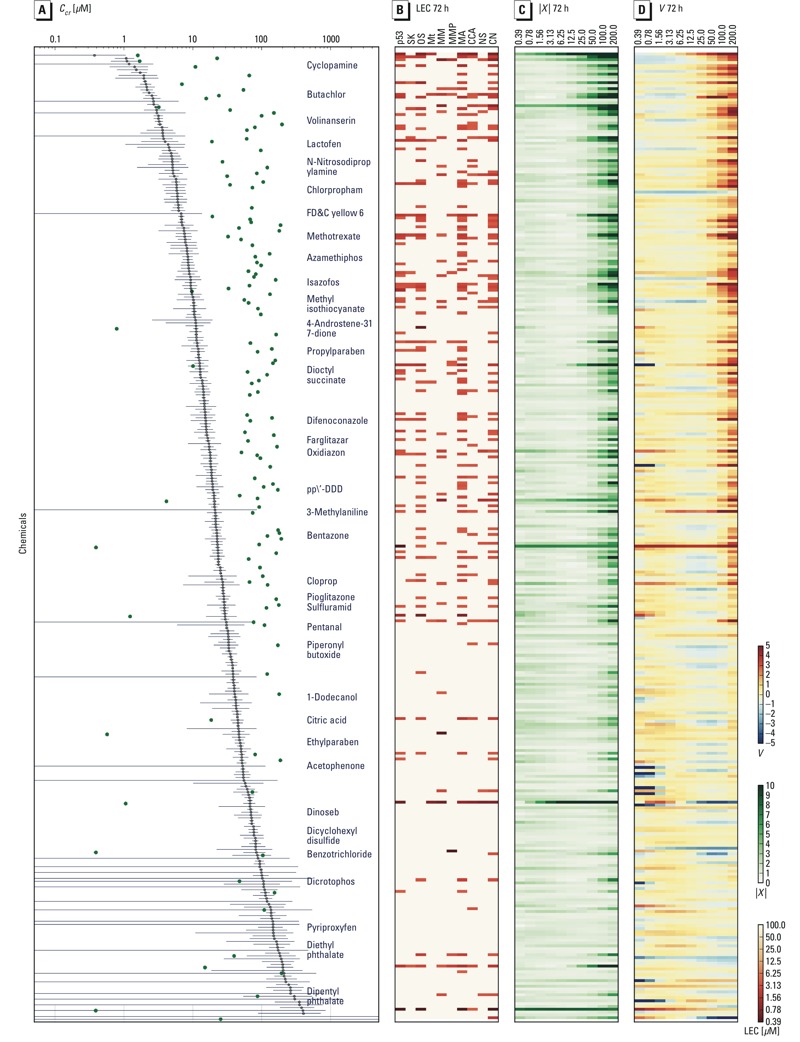
Critical concentrations (*C_cr_*) for 340 chemicals at 72 hr. Chemicals are sorted by *C_cr_* in descending order from top to bottom (*y*-axis), and each row shows the *C_cr_*, the lowest effect concentration (LEC), the scalar perturbation (**|*X*|**), and the velocity (*V*). (*A*) *C_cr_* s (μM) are indicated by points along the *x*-axis; the uncertainty is indicated by the gray line, the minimum LECs are green points, and select chemicals are labeled. (*B*) LEC (μM) across p53, SK (stress kinase), OS (oxidative stress), Mt (microtubules), MM (mitochondrial mass), mitochondrial membrane potential (MMP), mitotic arrest (MA), cell cycle arrest (CCA), nuclear size (NS), and cell number (CN). The LEC value is represented as no effect (pink), through saturation (red), as shown in the color bar on the right. (*C*) **|*X|*** as a heat map across concentrations (μM), where magnitude is represented by color saturation (values shown in color bar on the right). (*D*) *V* as a heat map across concentrations (μM) where *V *> 0 (reds), *V *< 0 (blues), and *V *= 0 (white).

We selected the 336 chemicals that elicited tipping points in the HepG2 system to compare critical concentrations with lowest effect concentrations (LECs), and the results are presented in Figure 5. Of the 336 chemicals that produced tipping points, only 124 had an LEC across any of the 10 end points at 72 hr. On average, the *C*_cr_ was 13 times lower than the lowest LEC for 86% (106/124) of the chemicals, whereas the LEC was 6 times lower than the *C*_cr_ for 15% (18/124) of the chemicals. In general, the *C*_cr_ was between 5 and 15 times (25th and 75th percentiles, respectively) lower than the lowest LEC. The results of the resilience analyses for 967 chemicals, along with critical points, are given in Excel Table S3.

## Discussion

From these results, we can conclude that HCI can be used to identify *in vitro* cellular tipping points in response to chemical-induced perturbations. HCI has previously been used for screening to study the effects of chemicals on cellular systems (O’Brien et al. 2006; Abraham et al. 2008) and to profile molecular changes underlying cellular processes (Neumann et al. 2010; Held et al. 2010). Here, we analyzed time-course HCI data to investigate the dynamic response of HepG2 cells to 10 concentrations of 967 chemicals. The time-dependent perturbations of HepG2 cells were analyzed as state trajectories that described sequential perturbations in the system state as it adapted to chemical exposure. A novel computational approach was developed to analyze these trajectories by quantifying the dynamic response of the system across all chemical treatments. The quantity of the scalar perturbation was termed the “velocity” because it measured the rate at which the aggregate system state deviated from, or returned to, the normal state. We hypothesize that this velocity is a measure of system resilience and that it can be used to identify a dose-dependent transition in system recovery. We call this dose-dependent transition a “tipping point” and believe that it can be used as a point of departure in a high-throughput risk assessment context (Judson et al. 2011).

At the present time, toxicological tests are based on identifying apical adverse effects to define a point of departure for risk assessment. An adverse effect has been traditionally defined as a “biochemical, morphological or physiological change (in response to a stimulus) that either singly or in combination adversely affects the performance of the whole organism or reduces the organism’s ability to respond to an additional environmental challenge” (Lewis et al. 2002). Characterizing adverse effects using high-throughput assays is a key problem for toxicology in the 21st century (Keller et al. 2012). HCI can measure adaptive cell stress responses, albeit in a cell-autonomous context (Simmons et al. 2009). An adaptive response is a homeostatic process that is activated by the system to survive in a new environment without impairment of function (Keller et al. 2012). We believe that our analysis of trajectories and tipping points brings us a step closer to realizing the vision of 21st century toxicology by providing a framework to identify where “transition points occur between adaptive changes and adverse effects” (Keller et al. 2012). However, implementing this vision will require much more work with regard to interpreting the role of cell-autonomous adaptive responses in the context of pathways that lead to *in vivo* adverse outcomes (Boekelheide and Andersen 2010).

Biological systems have evolved adaptive mechanisms that allow them to maintain a constant internal environment despite variations in external conditions (Kitano 2004). A number of homeostatic control systems compensate for chemical-induced perturbations in cells. Cells possess diverse signaling pathways to sense state changes caused by reactive oxygen species (ROS), DNA adducts, protein denaturation, glutathione depletion, and other agents, and can activate feedback control processes, typically via genetic regulatory networks, to maintain their internal state (Simmons et al. 2009). As the concentration of a chemical rises and the intracellular state becomes increasingly perturbed, different feedback control mechanisms are incrementally activated and, potentially, overwhelmed. The complexity of these interconnected processes could explain why we observed dose-dependent transitions in the recovery of HepG2 cells. Dose-dependent transitions have been described in the mechanisms of toxicity for a number of chemicals (Slikker et al. 2004), but such effects have not been studied systematically in *in vitro* systems. Zhang et al. (2008) proposed a control–theoretic approach to modeling the action of anti-stress genetic regulatory networks in maintaining cell state and to further explain the observation of dose-dependent transitions in biological responses. Experimental evidence (Slikker et al. 2004), together with mathematical models (Zhang et al. 2008), supports the notion that there are dose-dependent transitions in some biological responses; however, identifying *in vivo* thresholds for toxicity is expected to be multifactorial (time- and concentration-dependent) and thus is extremely challenging.

Assessing the global state of a cellular system, which is defined by thousands of biological molecules, is a challenging problem; however, a relatively small number of pathways may be involved in responding to chemical-induced stress. A set of such stress-response pathways proposed by Simmons et al. (2009) includes oxidative stress, heat shock response, DNA damage response, hypoxia, endoplasmic reticulum (ER) stress, metal stress, inflammation, and osmotic stress. Our study included a subset of these stress responses, but we also considered mitochondrial, cytoskeletal, and cell cycle changes, which are relevant measures of cell health. There is also increasing evidence for the occurrence of cross-talk between stress-response pathways, which enhances the adaptive response of cells to environmental stressors (Simmons et al. 2009). Assuming a finite number of stress-response pathways, the amplification of stress responses by cross-talk, and considering the sensitivity of HCI, we believe that our study reasonably assessed the adaptation of HepG2 cells to 967 chemicals. In subsequent studies, we plan to extend our analysis to include additional stress-response pathways.

It is important to note that our results may be limited by the small number of time points used in this study. Because our analysis combined HCI data from two independent experiments [ToxCast™ Phase I (Judson et al. 2010) and Phase II], the 1-hr time point was only collected for a subset (308/967) of chemicals, primarily because of cost considerations, and our preliminary results indicated that the 1-hr time point was helpful for visualizing trajectories but not essential to the analysis and conclusions. The analysis of tipping points is based on the complete set of observations for all 967 chemicals at 24 and 72 hr. We recognize that a more fine-grained temporal resolution or additional time points may produce different results. In particular, some chemicals that produced time-dependent results but did not display recovery at 72 hr may exhibit recovery at later time points. We hope to evaluate the impact of additional time points on the analysis of trajectories and tipping points in subsequent work.

Another potential limitation is the cell system used in this study. HepG2 cells are an immortalized cell line with characteristics that differ from those of normal hepatocytes. For example, these cells easily proliferate in culture but have limited metabolic activity compared with primary hepatocytes (Abraham et al. 2008; O’Brien et al. 2006). The HepG2 cell model used in this study was a two-dimensional monoculture that does not reflect the complex cell-to-cell interactions present in intact organs that have multiple cell types. Therefore, it is quite possible that the trajectories produced by chemicals in this cell-autonomous model will be different from those produced in more complex cell-based systems. However, we fully expect that the general categories of observations and the quantitative approach developed in the present study will be transferable to other cellular systems.

Tipping points for chemical-induced toxicity are not a new concept; however, defining them theoretically and identifying them experimentally are challenging. Chemical-induced toxicity is believed to occur when adaptive pathways in biological systems are overwhelmed, and it usually occurs when the stressor causes perturbations that are sufficiently large (Krewski et al. 2010). The idea that biological systems have a homeostatic capacity implies the existence of tipping points. If biological tipping points can be quantified for a chemical, they could be used to estimate levels of chemical exposure that overwhelm this homeostatic capacity. We believe that our approach for analyzing tipping points of cellular systems is an initial step toward quantifying *in vitro* regions of safety for chemicals. In combination with sophisticated methods for quantitative *in vitro* to *in vivo* extrapolation (Wetmore 2015), cellular tipping points could be used as points of departure for high-throughput risk assessment. The real-world application of this method will require additional evaluation of our approach using more chemicals, cell-based models, time points, and end points.

## Conclusions

Our findings demonstrate the potential utility of time-course high-throughput, high-content biological assays for elucidating cellular phenotypic behaviors invoked by chemicals and for identifying tipping points of cellular systems. The number of chemicals used in this study and the range of cellular end points measured suggest that this analytical approach can be used to provide valuable information about the effects of new chemicals and about critical concentrations at which cellular responses fail to return to control levels. Our findings also underscore the importance of considering the temporal evolution of biological systems as a means of resolving adaptive changes that either lead to recovery or progress to cellular injury.

## Supplemental Material

(20.4 MB) PDF

(4.3 MB) ZIP
