# Supplementary material for: Using ToxCast™ Data to Reconstruct Dynamic Cell State Trajectories and Estimate Toxicological Points of Departure
Source: Environ Health Perspect. 2015 Oct 16;124(7):910–9. doi: 10.1289/ehp.1409029 (PMC4937847; doi:10.1289/ehp.1409029)
Supplement: (20.4 MB) PDF [file ehp.1409029.s001.acco.pdf]

Environ Health Perspect
DOI: 10.1289/ehp.1409029

Note to readers with disabilities: *EHP* strives to ensure that all journal content is accessible to all readers. However, some figures and Supplemental Material published in *EHP* articles may not conform to 508 standards due to the complexity of the information being presented. If you need assistance accessing journal content, please contact ehp508@niehs.nih.gov. Our staff will work with you to assess and meet your accessibility needs within 3 working days.

Supplemental Material

Using ToxCast™ Data to Reconstruct Dynamic Cell State Trajectories and Estimate Toxicological Points of Departure

Imran Shah, R. Woodrow Setzer, John Jack, Keith A. Houck, Richard S. Judson, Thomas B. Knudsen, Jie Liu, Matthew T. Martin, David M. Reif, Ann M. Richard, Russell S. Thomas, Kevin M. Crofton, David J. Dix, and Robert J. Kavlock

Table of Contents

Figure S1. Sample high-content images for select chemicals: (a) CCCP, (b) taxol, (c) butachlor, (d) fludioxonil and (e) fluazinam. Each panel shows raw images for each chemical as a hierarchical grid in which the stains/fluorochromes are organized as rows, followed by time (sub-rows), and concentrations are given in the columns. The first column from the left shows the negative control (DMSO) followed by increasing concentrations of the treatment chemical. In order to provide a more detailed view of the cell-level changes for concentrations and time points, each image shows four fields. The magnification of all images was 20x unless labeled otherwise. For some chemicals we could not obtain the raw images for all stains/fluorochromes, concentrations and times. Finally, because all figures were generated automatically using the database (and not manually) some of the images may contain artifacts.

Figure S2. Raw concentration and time response data for reference chemicals, taxol and CCCP. Panels (a) and (b) show concentration (x-axis) dependent raw responses (y-axis) in cell number (CN), nuclear size (NS), mitochondrial membrane potential (MMP), mitotic arrest (MA) and mitochondrial mass (MM) for CCCP and taxol, respectively. The Responses at 1, 24 and 72 h are shown in different colors (see legend). Raw data (labeled as, "+") and smoothed data are shown as curves (see text for additional information). Panels (c) and (d) show the time (x-axis) dependent raw responses (y-axis) across CN, NS, MMP, MA and MM for CCCP and taxol, respectively. The responses at different concentrations

are signified by colors (see legend). The mean and standard deviation of responses for DMSO are shown in green.

Figure S3. Concentration and time-dependent responses across microtubules (Mt), p53 activity (p53), oxidative stress (OS), mitotic arrest (MA), mitochondrial membrane, potential (MMP), cell cycle arrest (CCA), cell number (CN), stress kinase (SK), mitochondrial mass (MM) and nuclear size (NS) for (a) fludioxonil, (b) fluazinam, and (c) butachlor. Each graph shows the smoothed raw responses in the y-axis, and the time in hours along the x-axis. The responses for 10 treatment concentrations are shown in different colors (see legend). Finally, the mean and standard deviation of raw responses for each endpoint for the DMSO controls are shown in green.

Table S1. The algorithms used for extracting extracting cell level features from the raw images. From left to right, each row shows the cell level feature(s), the cell stain or fluorochrome used by the algorithm, the name of the algorithm, and the name of the BioApplication software.

Supplemental Code and Data Zip File

Excel Table S1. The lowest effect concentration (LEC) for 967 chemicals. Each row shows the LECs for one chemical at one time point across the ten high-content imaging endpoints. From left to right the first three columns show the ToxCast chemical identifier (hchem_id), the CAS number (chem_casrn), and the time (timeh) in hours (h). The remaining columns show the micromolar (μM) LEC value for p53, SK (stress kinase), OS (oxidative stress), Mt (microtubules), MM (mitochondrial mass), mitochondrial membrane potential (MMP), mitotic arrest (MA), cell cycle arrest (CCA), nuclear size (NS), and cell number (CN).

Excel Table S2. Perturbations of the HepG2 system produced by 967 chemicals across 10 high-content imaging (HCI) endpoints, 10 concentrations and different time points. Each row shows the concentration and time dependent perturbations produced by each chemical across the 10 HCI endpoints. From left to right the first four columns show the ToxCast chemical identifier (hchem_id), the chemical name (chem_name), the time (timeh) in hours (h) and the micromolar (μM) concentration (conc). The remaining columns show perturbations as log_2(fold change) values for each HCI endpoint including: p53, SK (stress kinase), OS (oxidative stress), Mt (microtubules), MM (mitochondrial mass), mitochondrial membrane potential (MMP), mitotic arrest (MA), cell cycle arrest (CCA), nuclear size (NS), and cell number (CN).

Excel Table S3. The HepG2 system tipping points and critical concentration (C_{cr}) analysis for all 967 chemicals at 72 h. Each row shows the mean and standard deviation of C_{cr} for each chemical along with results of the uncertainty analysis. From left to right the columns show the ToxCast chemical identifier (hchem_id), the chemical name (chem_name), the CAS number (chem_cas), the confidence in the tipping point (tip_pt_pcnt), the mean value of C_{cr} (C_crit_mn) and the standard deviation of C_{cr} (C_crit_sd). The last column on the right shows the overall result of the trajectory analysis (traj_analysis), which has one of three possible values: recovered, tipping point and uncertain.

Figure S1. Sample high-content images for select chemicals: (a) CCCP, (b) taxol, (c) butachlor, (d) fludioxonil and (e) fluazinam. Each panel shows raw images for each chemical as a hierarchical grid in which the stains/fluorochromes are organized as rows, followed by time (sub-rows), and concentrations are given in the columns. The first column from the left shows the negative control (DMSO) followed by increasing concentrations of the treatment chemical. In order to provide a more detailed view of the cell-level changes for concentrations and time points, each image shows four fields. The magnification of all images was 20x unless labeled otherwise. For some chemicals we could not obtain the raw images for all stains/fluorochromes, concentrations and times. Finally, because all figures were generated automatically using the database (and not manually) some of the images may contain artifacts.

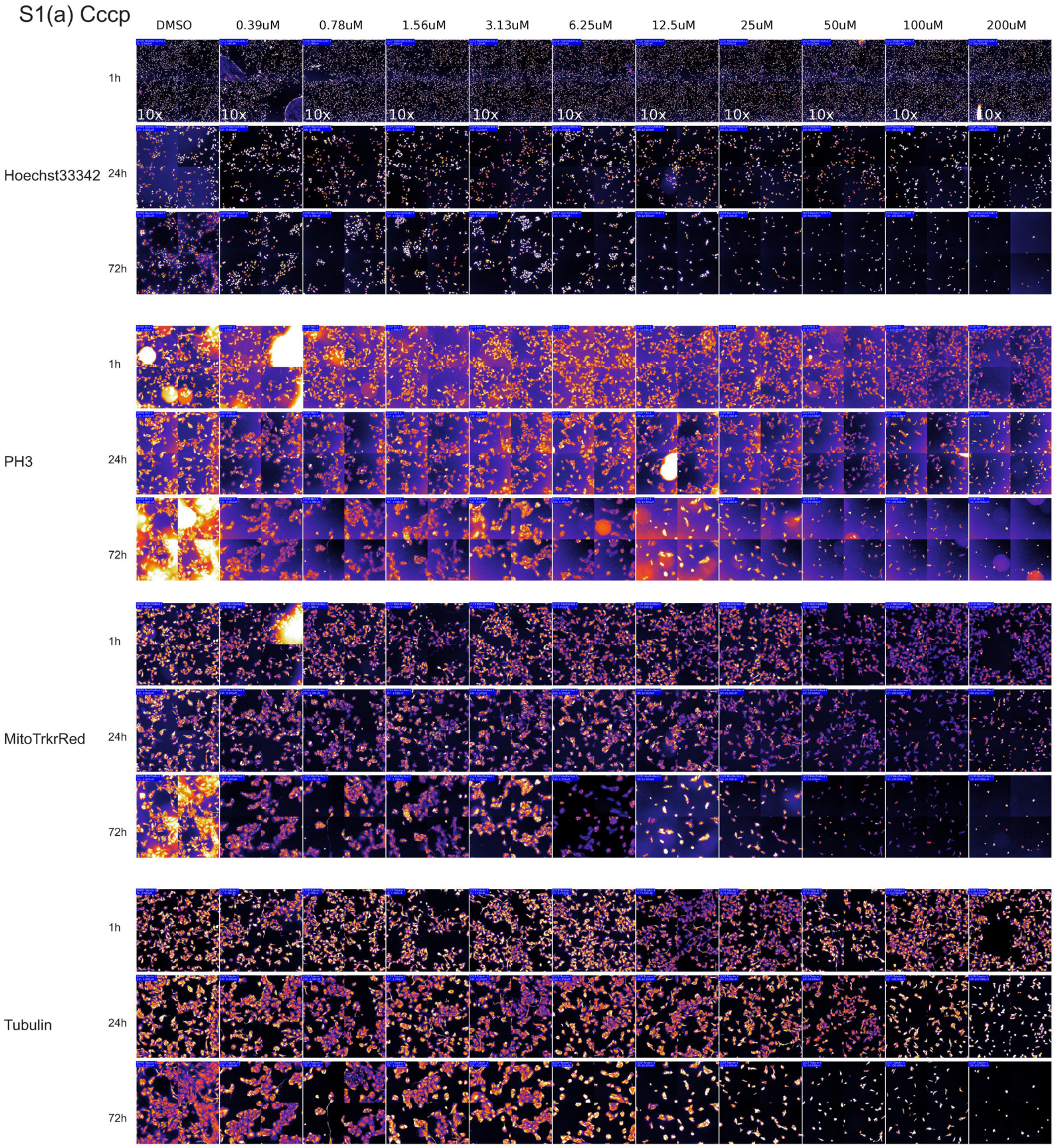

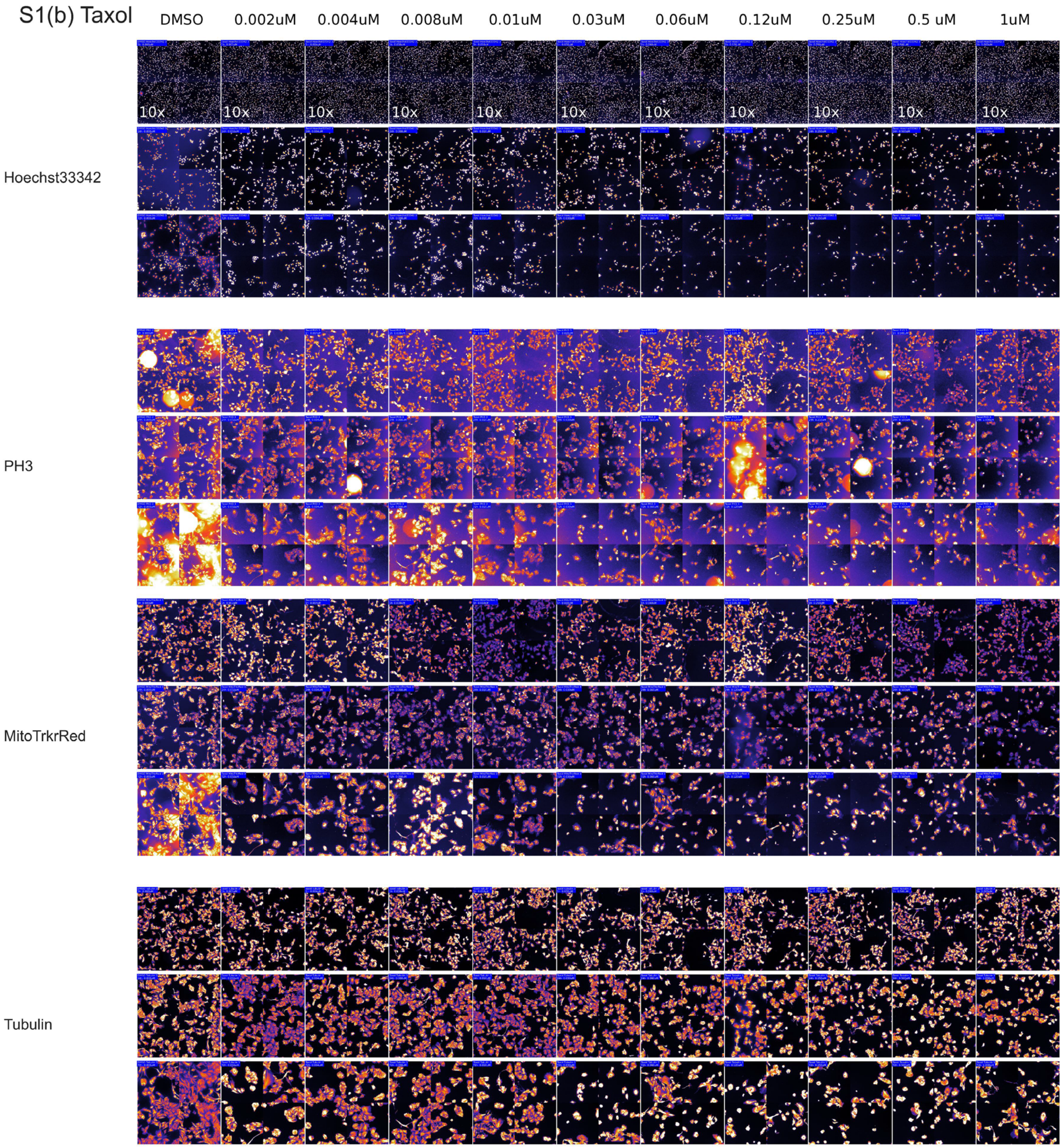
S1(b) Taxol
DMSO
0.002uM
0.004uM
0.008uM
0.01uM
0.03uM
0.06uM
0.12uM
0.25uM
0.5 uM
1uM
10x
Hoechst33342
PH3
MitoTrkrRed
Tubulin

S1(c) Butachlor

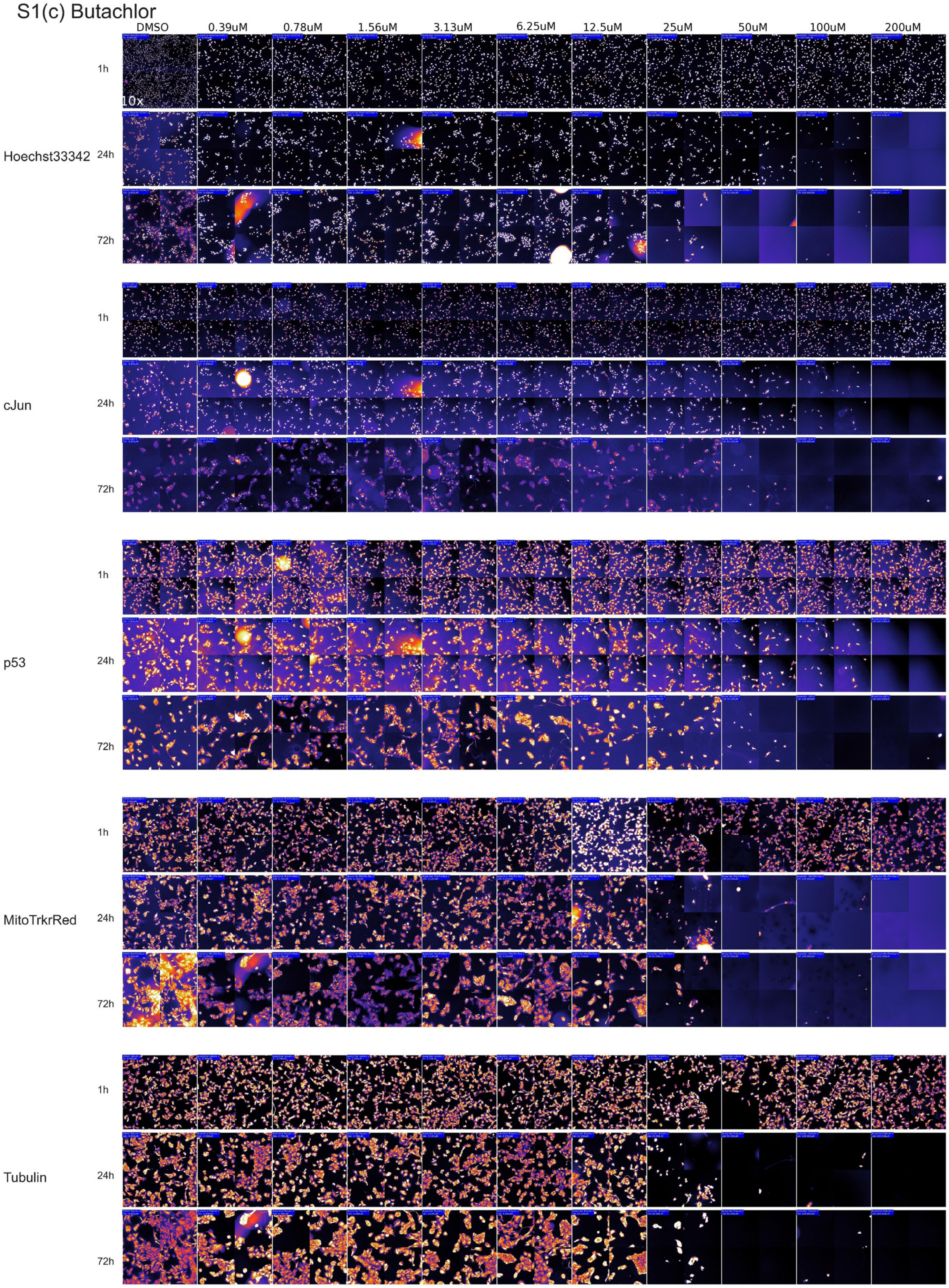

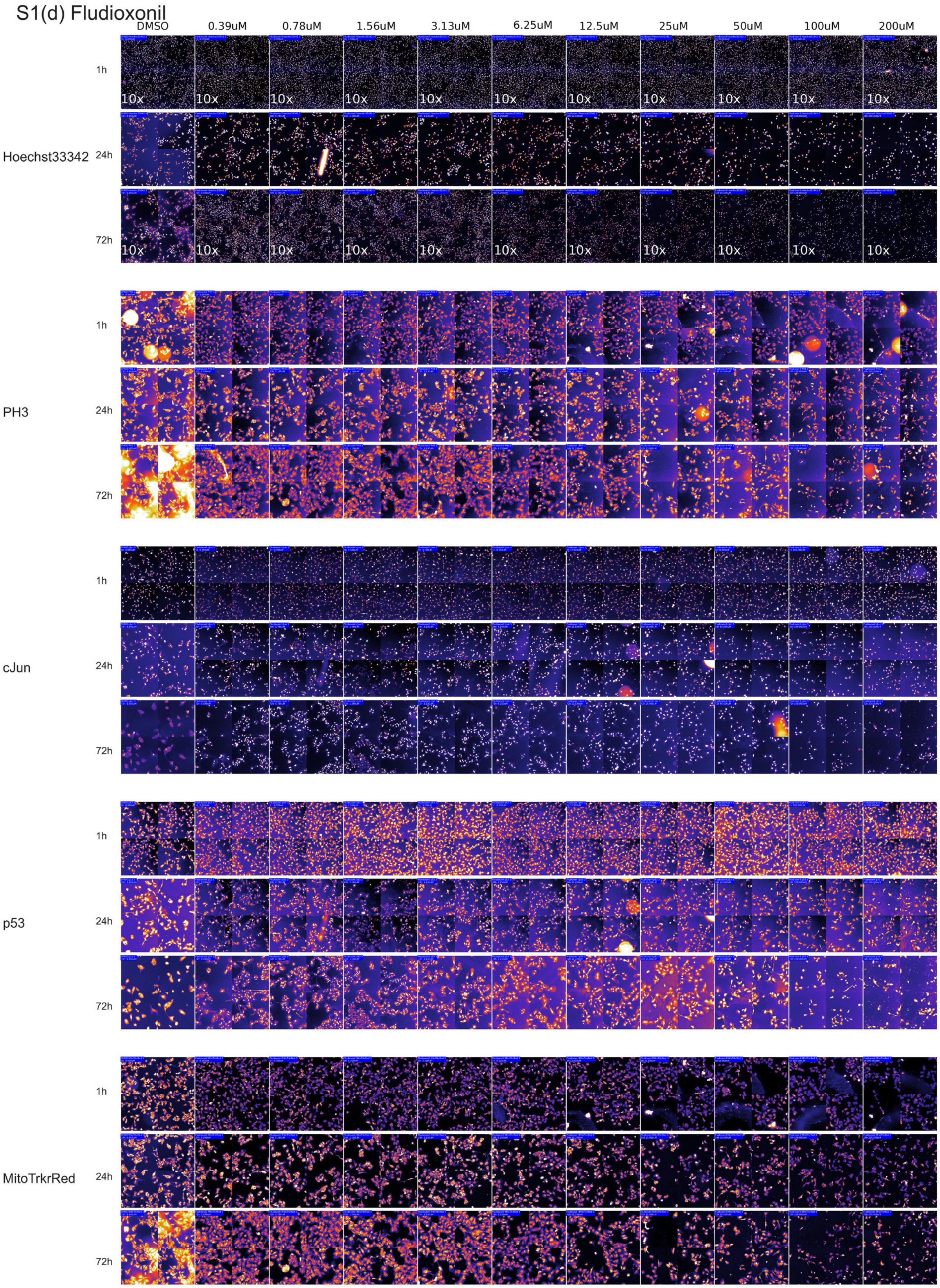
S1(d) Fludioxonil
DMSO
0.39uM
0.78uM
1.56uM
3.13uM
6.25uM
12.5uM
25uM
50uM
100uM
200uM
Hoechst33342
PH3
cJun
p53
MitoTrkrRed
1h
24h
72h
10x

S1(e) Fluazinam

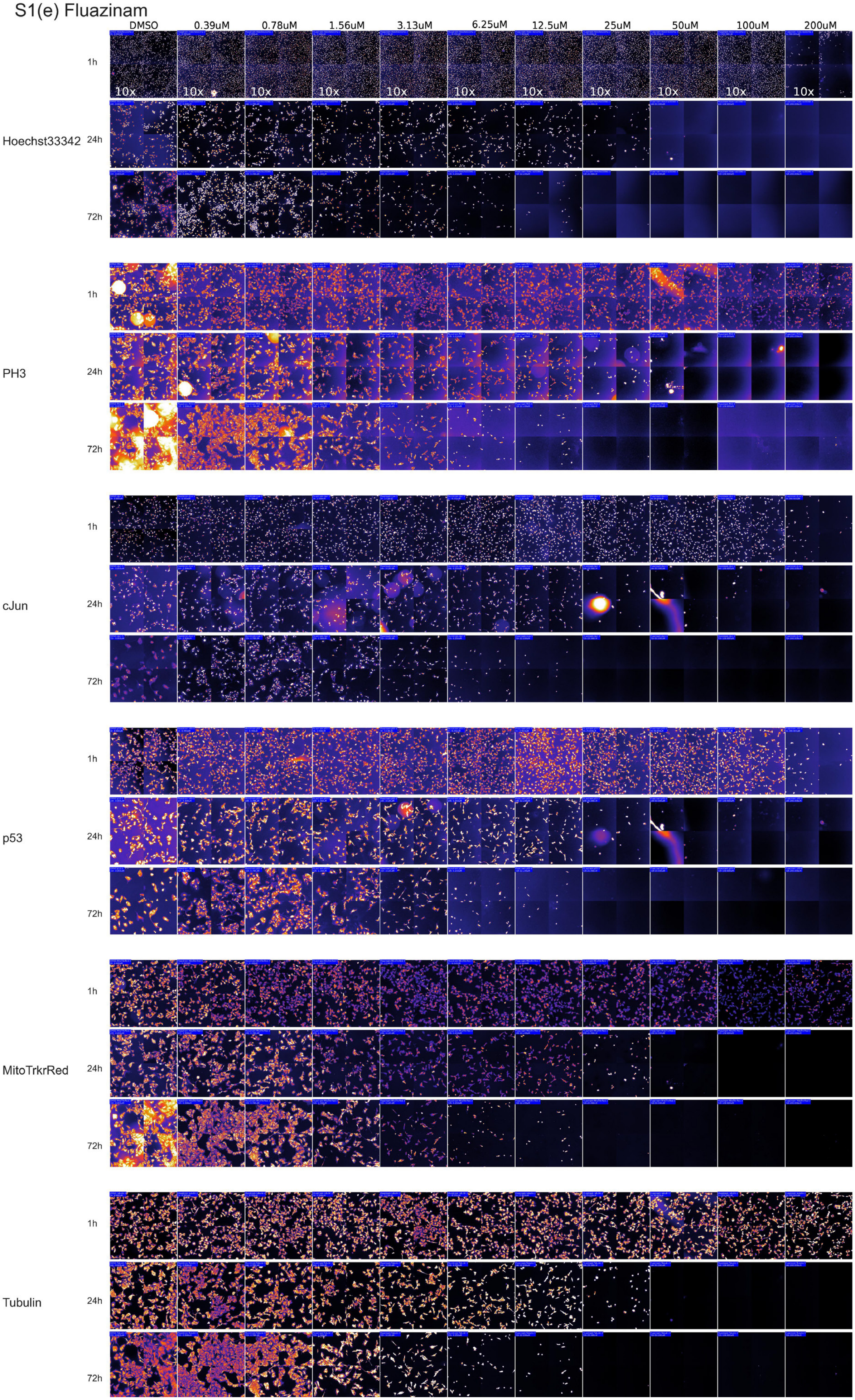

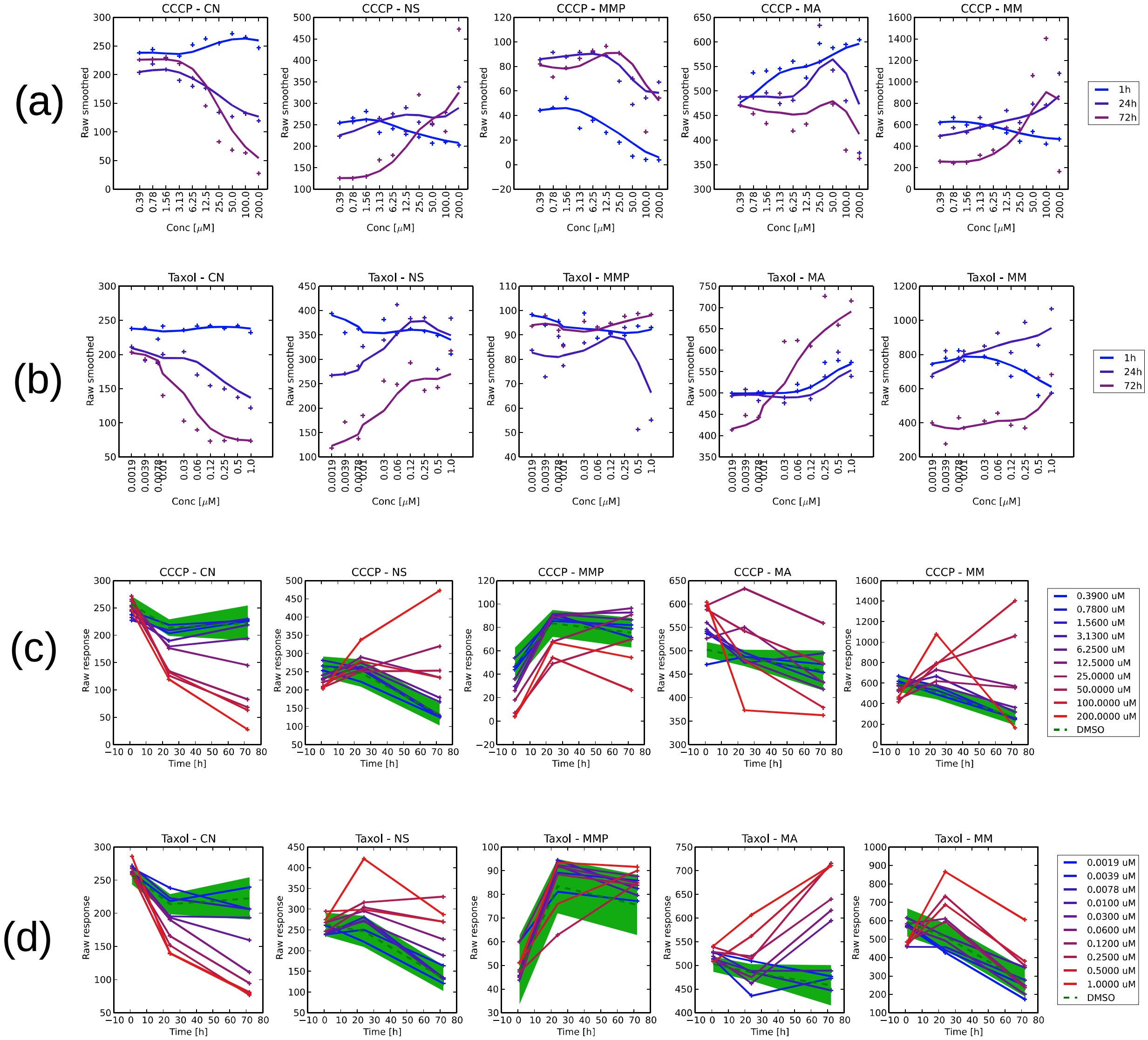

Figure S2. Raw concentration and time response data for reference chemicals, taxol and CCCP.

Panels (a) and (b) show concentration (x-axis) dependent raw responses (y-axis) in cell number (CN), nuclear size (NS), mitochondrial membrane potential (MMP), mitotic arrest (MA) and mitochondrial mass (MM) for CCCP and taxol, respectively. The Responses at 1, 24 and 72 h are shown in different colors (see legend). Raw data (labeled as, "+") and smoothed data are shown as curves (see text for additional information).

Panels (c) and (d) show the time (x-axis) dependent raw responses (y-axis) across CN, NS, MMP, MA and MM for CCCP and taxol, respectively. The responses at different concentrations are signified by colors (see legend). The mean and standard deviation of responses for DMSO are shown in green.

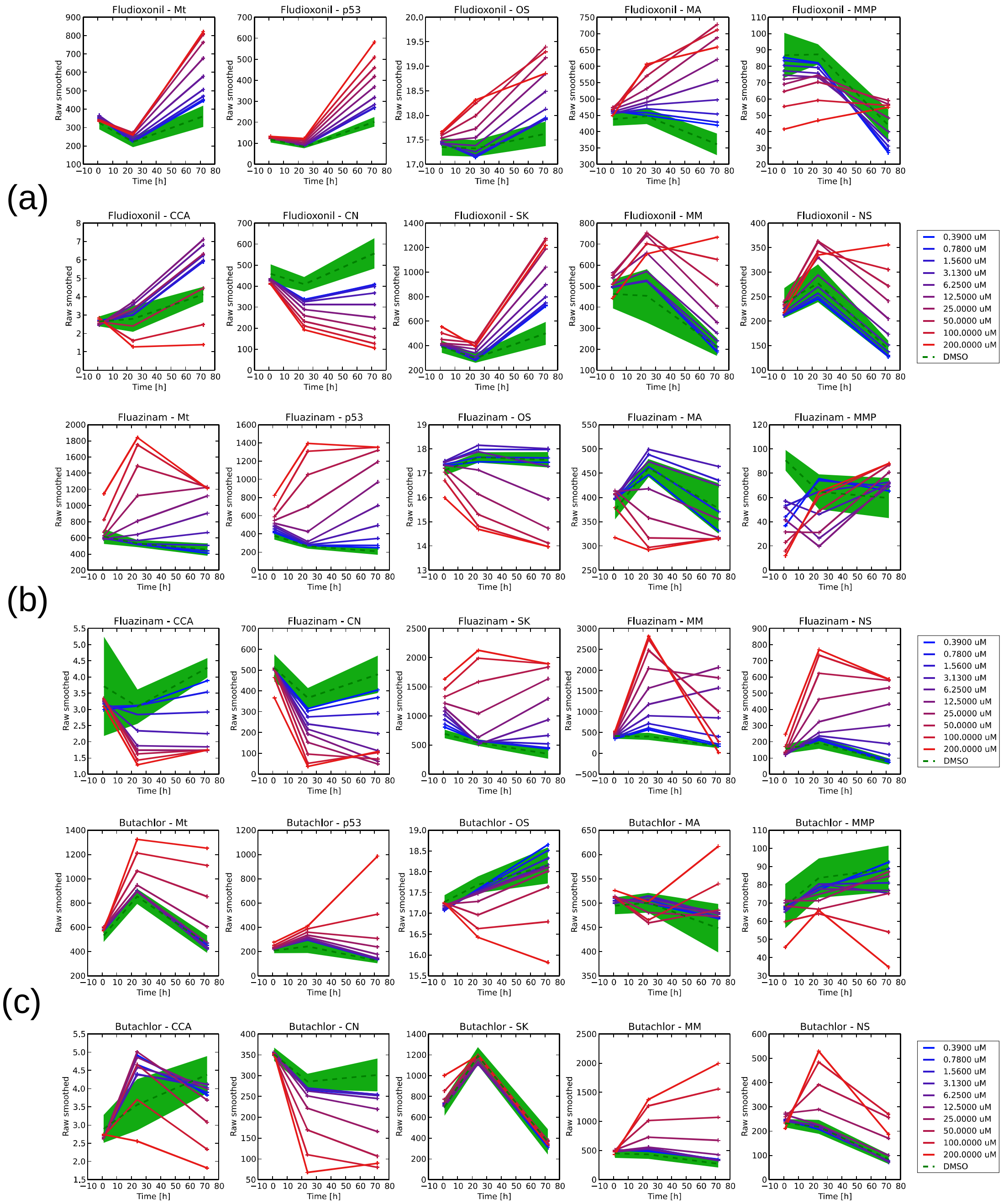

Figure S3. Concentration and time-dependent responses across microtubules (Mt), p53 activity (p53), oxidative stress (OS), mitotic arrest (MA), mitochondrial membrane, potential (MMP), cell cycle arrest (CCA), cell number (CN), stress kinase (SK), mitochondrial mass (MM) and nuclear size (NS) for (a) fludioxonil, (b) fluazinam, and (c) butachlor. Each graph shows the smoothed raw responses in the y-axis, and the time in hours along the x-axis. The responses for 10 treatment concentrations are shown in different colors (see legend). Finally, the mean and standard deviation of raw responses for each endpoint for the DMSO controls are shown in green.

Table S1. The algorithms used for extracting extracting cell level features from the raw images. From left to right, each row shows the cell level feature(s), the cell stain or fluorochrome used by the algorithm, the name of the algorithm, and the name of the BioApplication software.

Endpoint	Stain/ Fluorochrome	Algorithm	Bioapplication Software
Cell Number<br>Cell Cycle Arrest<br>Nuclear Size	Hoechst 33342	CellCountPerField<br>CellCountRatio_2N_4N_Ch1<br>MEAN_ObjectSizeCh1	Cell Cycle<br>Compartmental Analysis
Oxidative Stress[a]	phospho-Histone H2A.x	MEAN_CircAvgIntenCh2	Compartmental Analysis
Stress Kinase Activation	cJun Phosphorylation	MEAN_CircAvgIntenCh3	Compartmental Analysis
p53	p53 activation	MEAN_CircAvgIntenCh4	Compartmental Analysis
Mitochondrial Membrane Potential	MitoTracker Red	%HighRingSpotAvgIntenCh3	Compartmental Analysis
Mitochondrial Mass	MitoTracker Red	MEAN_RingSpotTotalAreaCh3	Compartmental Analysis
Mitotic arrest	H3 Phosphorylation	MEAN_CircAvgIntenCh2	Compartmental Analysis
Microtubule	Tubulin-microtubule stability	MEAN_CircAvgIntenCh4	Compartmental Analysis

a.Our findings about the effects of chemicals on H2AX activity do not imply that these chemicals are DNA damaging agents (none of the chemicals considered in this study are mutagenic). Increased levels of reactive oxygen species produced by chemicals can result in DNA damage and cause H2AX phosphorylation. Hence, we have chosen to allude to this assay as a measure of oxidative stress.

Excel Table S1. The lowest effect concentration (LEC) for 967 chemicals. Each row shows the LECs for one chemical at one time point across the ten high-content imaging endpoints. From left to right the first three columns show the ToxCast chemical identifier (hchem_id), the CAS number (chem_casrn), and the time (timeh) in hours (h). The remaining columns show the micromolar (μM) LEC value for p53, SK (stress kinase), OS (oxidative stress), Mt (microtubules), MM (mitochondrial mass), mitochondrial membrane potential (MMP), mitotic arrest (MA), cell cycle arrest (CCA), nuclear size (NS), and cell number (CN).

Excel Table S2. Perturbations of the HepG2 system produced by 967 chemicals across 10 high-content imaging (HCI) endpoints, 10 concentrations and different time points. Each row shows the concentration and time dependent perturbations produced by each chemical across the 10 HCI endpoints. From left to right the first four columns show the ToxCast chemical identifier (hchem_id), the chemical name (chem_name), the time (timeh) in hours (h) and the micromolar (μM) concentration (conc). The remaining columns show perturbations as log_2(fold change) values for each HCI endpoint including: p53, SK (stress kinase), OS (oxidative stress), Mt (microtubules), MM (mitochondrial mass), mitochondrial membrane potential (MMP), mitotic arrest (MA), cell cycle arrest (CCA), nuclear size (NS), and cell number (CN).

Excel Table S3. The HepG2 system tipping points and critical concentration (C_{cr}) analysis for all 967 chemicals at 72 h. Each row shows the mean and standard deviation of C_{cr} for each chemical along with results of the uncertainty analysis. From left to right the columns show the ToxCast chemical identifier (hchem_id), the chemical name (chem_name), the CAS number (chem_cas), the confidence in the tipping point (tip_pt_pcnt), the mean value of C_{cr} (C_crit_mn) and the standard deviation of C_{cr} (C_crit_sd). The last column on the right shows the overall result of the trajectory analysis (traj_analysis), which has one of three possible values: recovered, tipping point and uncertain.
